# The current socioeconomic and regulatory landscape of immune effector cell therapies

**DOI:** 10.3389/fmed.2024.1462307

**Published:** 2024-12-04

**Authors:** Chiranjeevi Sainatham, Devvrat Yadav, Aravind Dilli Babu, Jayanth Reddy Tallapalli, Sai Gautham Kanagala, Evgenii Filippov, Franco Murillo Chavez, Nausheen Ahmed, Forat Lutfi

**Affiliations:** ^1^Department of Internal Medicine, Sinai Hospital of Baltimore, Baltimore, MD, United States; ^2^Division of Infectious Diseases, Department of Internal Medicine, University of South Florida, Tampa, FL, United States; ^3^Department of Internal Medicine, New York Medical College/Metropolitan Hospital Center, New York, NY, United States; ^4^Department of Hematologic Malignancies and Cellular Therapeutics, University of Kansas Medical Center, Kansas City, KS, United States

**Keywords:** CAR T-cell therapy, immune effector cell therapy, regulatory environment for cellular therapeutics, bispecific antibodies (BsAbs), TILs (tumor infiltrating lymphocytes)

## Abstract

Immune cell effector therapies, including chimeric antigen receptor (CAR)-T cells, T-cell receptor (TCR) T cells, natural killer (NK) cells, and macrophage-based therapies, represent a transformative approach to cancer treatment, harnessing the immune system to target and eradicate malignant cells. CAR-T cell therapy, the most established among these, involves engineering T cells to express CARs specific to cancer cell antigens, showing remarkable efficacy in hematologic malignancies like leukemias, B-cell lymphomas, and multiple myeloma. Similarly, TCR-modified therapies, which reprogram T cells to recognize intracellular tumor antigens presented by major histocompatibility complex (MHC) molecules, offer promise for a range of solid tumors. NK-cell therapies leverage NK cells’ innate cytotoxicity, providing an allogeneic approach that avoids some of the immune-related complications associated with T-cell-based therapies. Macrophage-based therapies, still in early stages of the development, focus on reprogramming macrophages to stimulate an immune response against cancer cells in the tumor microenvironment. Despite their promise, socioeconomic and regulatory challenges hinder the accessibility and scalability of immune cell effector therapies. These treatments are costly, with CAR-T therapies currently exceeding $400,000 per patient, creating significant disparities in access based on socioeconomic status and geographic location. The high manufacturing costs stem from the personalized, labor-intensive processes of harvesting, modifying, and expanding patients’ cells. Moreover, complex logistics for manufacturing and delivering these therapies limit their reach, particularly in low-resource settings. Regulatory pathways further complicate the landscape. In the United States., the Food and Drug Administrations’ (FDA) accelerated approval processes for cell-based therapies facilitate innovation but do not address cost-related barriers. In Europe, the European Medicines Agency (EMA) offers adaptive pathways, yet decentralized reimbursement systems create uneven access across member states. Additionally, differing regulatory standards for manufacturing and quality control worldwide pose hurdles for global harmonization and access. To expand the reach of immune effector cell therapies, a multipronged approach is needed—streamlined regulatory frameworks, policies to reduce treatment costs, and international collaborations to standardize manufacturing. Addressing these socioeconomic and regulatory obstacles is essential to make these life-saving therapies accessible to a broader patient population worldwide. We present a literature review on the current landscape of immune effector cell therapies and barriers of access to currently approved standard of care therapy at various levels.

## Graphical abstract

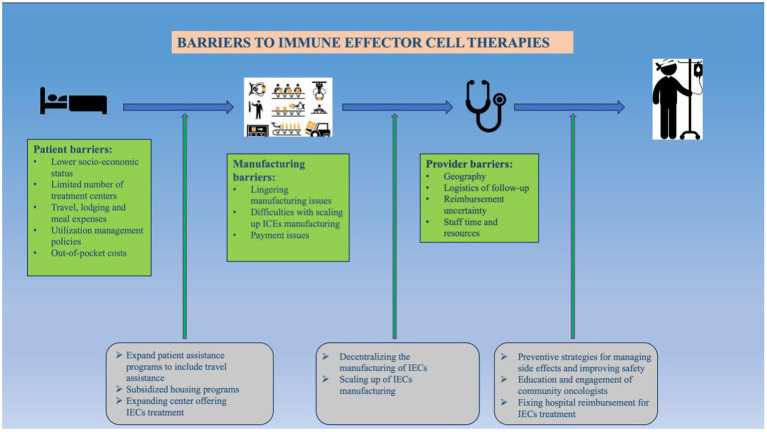

## Introduction

Cancer stands as a formidable global public health challenge, ranking as the second leading cause of mortality in the United States. As projected by the American Cancer Society for 2023, an alarming 1,958,310 new cancer cases and 609,820 cancer-related deaths are anticipated in the United States alone (1). As a result of intrinsic genomic instability in cancer cells, this situation becomes even more severe, leading to an increased mutational burden, as well as the emergence of diverse subclonal lines with distinct characteristics, including invasion, proliferation, drug resistance, and the presentation of oncoantigens and neoantigens creating dire challenges for treatment options (2). Even though conventional cancer therapies, such as chemotherapy, radiation therapy, and hormone therapy, demonstrate efficacy, their impact is limited by side effects and the emergence of resistance during treatment (3). This amplifies the need for alternative approaches, prompting a search for innovative strategies to complement existing therapies. During the nineteenth century, Wilhelm Busch and Friedrich Fehleisen reported spontaneous tumor regressions following *Streptococcus pyogenes*-induced erysipelas, an infection that stimulates the immune system. This incident led to the idea of using the immune system for cancer treatment. In furthering this concept, William Coley is often referred to as the “Father of Cancer Immunotherapy” (4). The advent of cell therapies, particularly immune cell therapies, has ushered in a paradigm shift in drug development. Cancer immunotherapy has been designated “breakthrough of the year” by science in 2013 for its ability to target and eradicate malignant cells by exploiting the specificity and killing mechanisms of the immune system. There has been a notable advancement in immune cell therapies, which have shown clinically significant benefits in the treatment of cancer. By targeting cytokines, chemokines, and immune cells, immunotherapy makes a significant difference from traditional approaches, reshaping the tumor microenvironment (TME) and preventing cancer recurrence (5). Several studies have demonstrated a significant improvement in overall survival and a reduction in cancer recurrence with combinatorial approaches when immunotherapy is coupled with conventional chemoradiotherapy. This combined approach not only holds promise for enhanced treatment outcomes but also presents opportunities to elevate the quality of life for patients. While targeted and immune therapies continue to evolve, adoptive cell therapies (ACTs) have gained considerable attention in oncology. As a means of combating disease, these strategies involve infusion of lymphocytes, primarily autologous T-cells (6). A pinnacle in this arena is chimeric antigen receptor (CAR)-T cell therapy, marking a major breakthrough in cancer care with the approval of tisagenlecleucel by the Food and Drug Administration (FDA) in 2017 (7). Using autologous T-cells that have been extracted from the patient’s peripheral blood and given increased specificity and killing effectiveness against the patient’s malignant cells via a viral vector, CAR-T cell therapy helps remove tumors by reintroducing the modified immune cells into the patient (8). By April 2023, six CAR T cell therapies had received FDA approval, demonstrating unprecedented efficacy, particularly in patients with B-cell malignancies and multiple myeloma (9). Other forms of immune effector therapies that are currently approved and with further clinical trials underway are bispecific T-cell engagers (BiTEs), and tumor infiltrating lymphocytes (TILs) among others. To better understand the current use and potential future use of immune effector cell (IEC) therapy, we present a review of socio-economical, logistical, and pertinent factors influencing access and use of these therapies in the United States and around the world.

## Clinical applications

### Success stories and breakthrough in hematological malignancies

CAR-T cell therapy has transformed the treatment landscape for several hematologic malignancies that previously lacked suitable therapeutic options. Since 2017, FDA has approved six CAR-T therapies, including axicabtagene ciloleucel (axi-cel) for relapsed or refractory (r/r) Diffuse Large B-cell Lymphoma (DLBCL) and (r/r) follicular lymphoma (FL); tisagenlecleucel (tisa-cel) for (r/r) Acute Lymphoblastic Leukemia (ALL) in those under age 25 years, follicular lymphoma (FL), and DLBCL; lisocabtagene maraleucel (liso-cel) for (r/r) DLBCL, high grade FL, mantle cell lymphoma (MCL), and chronic lymphocytic leukemia (CLL); brexucabtagene autoleucal (brex-cel) for (r/r) mantle cell lymphoma (MCL) and ALL; idecabtagene vicleucel (ide-cel), and cilatacabtagene autoleucel (cila-cel) for (r/r) multiple myeloma (MM). Notably, four of these CAR-T cell products target anti-cluster of Differentiation (CD)19, while the two most recent ones focus on B-cell maturation antigen (BCMA) and have achieved rapid and durable responses in patients with R/R- ALL, FL, MCL, DLBCL, CLL, and MM (10–18).

In spite of the notable success achieved in treating B-cell malignancies with anti-CD19 CAR-T therapy, the challenge of relapse in patients who attain complete remission persists. This issue is particularly pronounced, with observed relapse rates ranging from 40 to 60%, notably in DLBCL (14, 17, 19, 20). The development of second-generation CAR-T featuring costimulatory domains (CD28, 4-1BB, and OX40) has resulted in substantial cell expansion and heightened tumor activity (21). Recent studies indicate the promising impact of second-generation anti-CD20 CAR-T cells with 4-1BB in individuals with relapsed/refractory CD20+ DLBCL (22). Furthermore, emerging therapeutic avenues, such as IgG-like bispecific antibodies, have garnered attention and gained approval for treating DLBCL (23). Similarly, with FDA approval and adoption of anti-BCMA CAR-T cell therapy for r/r MM, concern of antigen escape has prompted the exploration of various other tumor-specific antigens (TSAs) like CD138 (12–16). Presently, multiple clinical trials utilizing the second generation of anti-CD138 CAR-T cell therapy are in progress, revealing favorable tolerability and efficacy in this setting (24).

Further investigations have been done to broaden the application of CAR-T to other hematological malignancies. Acute Myeloid Leukemia (AML), the most prevalent acute adult leukemia, unfortunately, has not achieved comparable success with CAR-T cell therapy as observed in ALL, primarily due to antigenic heterogeneity and leukemic stem cells. Recently identified TSAs, such as CD123, CD33, and CD38 expressed on leukemic stem cells, present a challenge given their expression on hematopoietic stem cells as well. Unfortunately, targeting these TSAs has been associated with prolonged myelosuppression and a risk of toxicity (25–27). To address this, innovative strategies have been implemented within the CAR structure, including the development of rapidly switchable universal (uni) anti-CD123 CAR-Ts. UniCAR-T maintains complete anti-leukemic efficacy while ensuring prompt controllability, thereby improving the safety and adaptability of CD123-directed immunotherapy. The safety and efficacy of UniCAR-T, in conjunction with CD123, are presently undergoing evaluation in a phase I clinical trial (28). Additionally, the expression CD70 on AML blasts, coupled with its absence in normal myeloid cells, positions it as a promising target for AML treatment. The safety and efficacy of anti-CD70 CAR-T cell therapy are currently under study (29, 30).

Similarly, the influence of antigenic heterogeneity has been evident in other hematological malignancies, particularly highlighted by the absence of CD19 expression in Hodgkin Lymphoma (HL) and T-cell malignancies (11). This has prompted an exploration aimed at identifying various potential TSAs, including CD7, CD20, CD22, CD30, CD38, and CD138 (24, 31–33). CD30, universally expressed in classical HL, has recently been the focus of several clinical trials assessing the safety and efficacy of anti-CD30 CAR-T cell therapy in relapsed/refractory HL (31).

### Success stories and breakthrough in solid tumors

The notable successes of CAR-T therapy treatment in hematological malignancies strongly support the application of CAR-T therapy for treating solid tumors. However, the efficacy of CAR-T therapy in the context of solid tumors remains inconclusive and is hindered by various challenges such as the identification of TSAs, addressing the escape of tumor antigens, limited tumor cell trafficking and infiltration and the existence of an immunosuppressive tumor microenvironment. Despite the lack of antigen specificity and heterogeneity, recent research has achieved noteworthy breakthroughs in treating certain solid tumors (34). Subsequent research has identified several potential TSAs associated with solid tumor malignancies. Examples include human epidermal growth factor receptor-2 (HER2) expressed in certain sarcomas, IL-13Rα2 expression in glioblastoma multiforme (GBM), GD2 expression in neuroblastoma, epidermal growth factor receptor (EGFR) expression in non-small-cell lung carcinoma (NSCLC), breast, gastroesophageal colorectal cancers, and recurrent GBM, as well as Carcinoembryonic antigen (CEA) expression in colorectal cancer (CRC), lung, breast, and pancreatic cancers and claudin18.2, expression is noted in 70% of primary gastric cancers (35–44).

Claudin18.2, a TSA and a stomach-specific isoform of Claudin-18, is detected in 70% of primary gastric adenocarcinomas and their metastases (44). Recognized as a potential target for treating these malignancies, CT041, an anti-CLDN18.2 CAR-T cell product, has obtained Investigational New Drug (IND) clearance from the FDA for use in patients with CLDN18.2-expressing stomach, pancreatic, and gastroesophageal junction adenocarcinoma (42). The IND clearance was supported by findings from a phase-I trial (NCT03874897), where Claudin18.2 CAR-T cell therapy demonstrated an overall response rate (ORR) and disease control rate of 57.1 and 75.0%, respectively, in gastric cancer patients. The 6-month overall survival rate was reported at 81.2%, with no serious adverse events documented. This CAR-T cell therapy study yielded an ORR of 33%, a median progression-free survival (PFS) of 130 days, and a well-tolerated safety profile, devoid of serious adverse events (43). Similarly, positive outcomes were observed in a phase I/II clinical study (NCT00902044) involving the use of HER2 CAR-T cells for the treatment of 19 patients with HER2-positive sarcomas, including 16 osteosarcomas, 1 primitive neuroectodermal tumor, 1 Ewing sarcoma, and 1 protofibroblastic small round cell tumor. Notably, the 19 treated patients achieved a median overall survival of 10.3 months, ranging from 5.1 to 29.1 months, and there were no major adverse events reported (36).

Similarly, EGFR, a TSA, plays a crucial role in the development and progression of solid tumors and has emerged as a significant therapeutic target in various cancers such as NSCLC, breast, gastroesophageal, colorectal cancers, and GBM. In a phase-I clinical trial (NCT02209376), 10 patients with Recurrent EGFRvIII+ GBM were treated with EGFRvIII-engineered CAR-T cells, showing an anti-tumor effect with a median overall survival of approximately 8 months in all patients. Another phase-I clinical trial (NCT01869166) of EGFR CAR-T cell therapy in 11 patients with EGFR+ refractory/relapsed non-small cell lung cancer (NSCLC) demonstrated that 2 patients achieved a partial response, and 5 had stable disease for 2 to 8 months without severe toxicity (39). Furthermore, IL-13Rα2, a TSA, is highly expressed in GBM tumor cells but is seldom found in normal brain cells, making it a compelling target for CAR-T cell therapy in GBM. In a study conducted by Brown and colleagues (NCT02208362), the multi-dose treatment with IL-13Rα2 CAR-T cells led to complete tumor regression for almost 8 months in a patient with GBM (33). Additionally, GD2, a disialoganglioside, is highly expressed in neuroblastoma cells, serving as another intriguing target for CAR-T cells in GBM (38). In a phase-I clinical trial (NCT00085930) assessing the impact of GD2 CAR-T cells on 11 patients with neuroblastoma, complete remission was observed in three patients.

Similarly, CEA, a TSA, stands out as a promising target for CRC (44). A phase-I, escalating-dose trial of CAR-T cell therapy (NCT02349724) directed against CEA expressed in metastatic CRC revealed that 7 out of 10 patients maintained stable disease for up to 30 weeks, and 2 patients experienced tumor reduction with no reported adverse events (45). Furthermore, CD133, another TSA, is among the well-characterized markers of cancer stem cells (CSCs) in various tumor types, including hepatocellular carcinoma (HCC) (46). In a phase I/II clinical trial (NCT02541370), CD133 CAR-T cells were administered to 21 patients with advanced HCC, demonstrating antitumor efficacy with low treatment-related toxicity (40).

### Ongoing research and prospects of CAR cell therapy

Current research endeavors are focused on developing therapeutic strategies to address challenges such as antigen escape, enhance the regulation of CAR-T cell persistence, and improve the overall anti-tumor efficacy of CAR-T cell therapy. These efforts involve exploring combinatorial approaches, including the incorporation of immune-stimulatory molecules, to overcome obstacles presented by the immunosuppressive microenvironment. Additionally, considering the socioeconomic factors associated with CAR-T therapy, ongoing investigations are exploring alternative approaches such as UniCAR-T, CAR-NK, and CAR-M therapies (47).

Targeting distinct antigens stands out as a highly effective approach to counteract antigen-negative relapse. Demonstrating efficacy in reducing the risk of such relapse, dual-targeting CAR-T cells, designed to recognize two distinct target antigens, have been employed. Examples include bispecific CAR-T cells designed for B cell lymphoma/leukemia and A proliferation-inducing ligand (APRIL)-based CAR-T cells simultaneously targeting both BCMA and transmembrane activator and calcium modulator and cyclophilin ligand interactor (TACI) in multiple myeloma (48, 49). Moreover, the exploration of multi-targeted CAR-T cells has been undertaken, with preclinical studies showcasing the swift elimination of B cell lymphoma using tri-specific CD19-CD20-CD22-targeting CAR-T cells (50). In addition, the short duration of CAR-T cell persistence hinders their effectiveness against tumors and increases the risk of antigen-positive relapse. Various strategies are being explored to enhance CAR-T cell persistence, including optimizing the CAR-T cell construct, utilizing memory T-cells, and carefully determining the ratio of CD4 to CD8 CAR-T cells (51). Presently, CD28 and 4-1BB are the most prevalent costimulatory molecules in CAR-T cell products. Notably, research has demonstrated that 4-1BB co-stimulation can mitigate CAR-T cell exhaustion in comparison to CD28 co-stimulation (52–54). Interestingly, the combination of CD28 and 4-1BB has shown the potential to simultaneously amplify anti-tumor effects and prolong the persistence of CAR-T cells (52–54). Furthermore, various immune-stimulatory molecules, including specific cytokines or co-stimulatory molecules, have been shown to be pivotal in regulating the development and functionality of T cells. Examples include IL-7, IL-12, IL-15, IL-18, IL-21, and CD40L (55–58). Moreover, refining the CAR-T cell structure is possible through the adoption of fully humanized CARs. Humanized CAR-T cells offer the advantage of evading rejection by the host immune system, and they maintain effectiveness even in patients with R/R disease who have previously experienced failure with prior CAR-T therapy (59). In addition, the programmed cell death (PD)-1/PD ligand (PDL)-1 pathway plays a crucial role in inducing T-cell exhaustion, serving as a key mechanism for tumors to evade the immune response. Consequently, disrupting the interaction between PD-1 and PD-L1 can enhance the immune system’s ability to combat cancer cells. Significantly, PD-1 blockade has demonstrated substantial success across various tumor types, particularly in lymphoma. In a clinical trial involving 11 NHL patients, 45.5% of individuals achieved complete responses (CRs) with this combined therapy, and the associated toxicities were well-tolerated (60, 61).

Manufacturing CAR-T cells is labor-intensive and costly, which hinders their widespread adoption (62). Due to the current production cycle, which takes 2 weeks or more, highly proliferative malignancies may progress rapidly in this period (63). In light of this, a new type of CAR-T cell therapy has gained attention: uniCAR-T. CAR-T cells derived from healthy donors are allogeneic instead of autologous CAR-T cells, which are derived from the same patient’s T-lymphocytes. In spite of their similar killing mechanism, uniCAR-T cells differ in their manufacturing processes, costs, safety considerations, and potential applications (64). A significant challenge to the realization of this therapy is graft-versus-host disease (GVHD), and potential toxicities like increased cytokine release syndrome (CRS) cannot be ruled out (65). A clinical trial (NCT01864889) reported grade 2–3 GVHD in two patients 4 weeks after donor-derived CAR-T cell infusions (66).

The ANTLER clinical trial recently published results on the use of readily available, off-the-shelf anti-CD19 CAR T-cell therapy with PD-1 knockout. Their partial HLA matching strategy yielded remarkable outcomes, with zero cases of GVHD in a test population of 46.

In contrast to CAR-T cells, chimeric antigen receptor NK (CAR-NK) cells target NK cells, another key component of the immune system that plays both an innate and adaptive role. NK cells function independently of major histocompatibility complex (MHC) and do not cause GVHD (47). In a clinical trial (NCT03056339) targeting CD19-positive lymphoid tumors, NK cells were transduced to express genes encoding anti-CD19 CAR, IL-15, and inducible caspase 9 as a safety switch. Out of the 11 treated patients, 8 (73%) responded, and 7 (64%) achieved complete remission. Importantly, there were no cases of CRS or neurotoxicity, and no apparent increase in inflammatory cytokines or GVHD was observed with this HLA-mismatched CAR-NK product (67). This initial study underscores the safety advantages of CAR-NK cells in universal cell therapy.

Furthermore, induced pluripotent stem cells (iPSCs) are currently a subject of intensive research, given their capacity for boundless self-renewal and differentiation into terminal cells. This includes T and NK cells, showcasing antitumor activity. Recent investigations have highlighted that CAR-NK cells generated from iPSCs not only significantly inhibited tumor growth but also extended survival in an ovarian cancer xenograft model (62, 68). Additionally, gene engineering has endowed macrophages with sustained proinflammatory phenotype (M1) and antigen-specific phagocytosis (38, 69). In two xenograft mouse models, CAR macrophages (CAR-M) targeting solid tumor antigens mesothelin or HER2 reduced tumor burden and prolonged overall survival, preliminarily proving its feasibility in solid tumors (69, 70).

Additionally, genetic engineering using CARs has conferred a persistent proinflammatory phenotype (M1) and antigen-specific phagocytosis to macrophages (69). In recent experiments involving two xenograft mouse models, CAR-M designed to target solid tumor antigens, specifically mesothelin or HER2, resulted in reduced tumor burden and extended overall survival. This provides initial evidence supporting the feasibility of utilizing CAR-M in the context of solid tumors (70).

### Other emerging cellular therapies

#### BITEs

Bispecific T-cell engagers (BiTEs) are a class of immunotherapy drugs that uses the antibodies to help the immune system fight cancer cells. BiTEs are made of two antibody arms that bring T cells and cancer cells together by binding to targeted antigens on these cells. The CD19-targeting BiTE (blinatumomab) was the first to be approved for cancer therapy after showing remarkable response rates of almost 70% in CD19-positive, relapsed/refractory (R/R) hematological malignancies. So far, there are 12 FDA-approved BiTEs since blinatumomab’s approval in 2014 with over 50 products in clinical trials.

Compared to CAR-T, BiTEs are easily manufactured. It has advantages, such as BiTE-mediated T-cell activation being independent of the TCR-MHC interaction, as most immune cell-engaging bispecific antibodies act by binding the CD3ε subunit on T-cells and a TAA on the tumor cell to form a cytolytic synapse. This bypasses the need for MHC presentation, directly triggering activation signaling leading to the release of the pore-forming perforin and cytotoxic granzyme-B (GzmB) and, ultimately, apoptosis of the target cell (71). In addition, BiTEs initiate bystander tumor cell killing and overcome immunosuppression by redirecting regulatory T-cells (Tregs). However, for BiTEs to be effective, having a target antigen is essential, or cell lysis was observed when treating cells or tumors lacking the target antigen (72).

Apart from hematological malignancies, Tebentefusp is a bispecific protein targeting gp100, which recently obtained FDA approval in January 2022 after a phase III trial showed a survival benefit for HLA-A*02:01–positive patients with metastatic uveal melanoma, increasing 1-year survival from 59 to 73% (73). Various clinical trials are being undertaken in solid tumors, such as Glioblastoma via EGFRvIII targeting BiTE (NCT04903795) and T-cell-bispecific antibody (TCB) targeting EGFRvIII (74). Although BiTEs appears promising, adverse effects persist and are either related to T-cell activation and cytokine secretion, culminating in CRS with or without neurotoxicity, or on-target off-tumor toxicity when the target antigen is expressed in normal tissues (75). Furthermore, challenges, such as antigen loss in T-cell engaging therapy, occur in about 30% of patients in studies of blinatumomab and account for one mechanism of treatment failure (76). The method of delivery of bispecific antibodies influences pharmacokinetics, the ability to penetrate tumor tissue fully, and the risk of systemic toxicity to which various strategies are being experimented such as DNA launched bispecific T-cell engagers (dBiTE), bispecific antibody armed activated T-cells (BATs), BiTE-secreting genetically engineered macrophages (GEMs), oncolytic viruses expressing bispecific antibodies. Pascual-Pasto et al. showcased an *in vitro* model where T-cells could be engineered to express a GPC2 CAR using a lentiviral vector and secrete a bispecific innate immune cell engager (or BiCE) that targets GD2 and FcγRIIIa (or CD16a) to activate bystander NK-cells and macrophages in the TME to facilitate antitumor innate immunity in neuroblastoma (77).

### Tumor infiltrating lymphocytes

TILs are polyclonal and are not genetically modified, as they are cultured directly from the TME, in contrast to CAR-T, which is specific to an antigen and genetically engineered. TILs are an adoptive cell therapy with infusion of these massively expanded, unmodified autologous T-cells as a personalized immunotherapy. This process is combined with non-myeloablative chemotherapy for preconditioning and high-dose interleukin-2 (IL-2) post-infusion to support the expansion and activation of TILs. This strategy has shown success in advanced melanoma, leading to FDA approval of lifileucel (Amtagvi) in 2024 (78). Despite the successes at individual centers, the relatively complex and prolonged manufacturing process and quality of the tumor specimen procured limited the widespread implementation of the TILs. Even with an optimized TIL product, treatment-associated toxicity is a concern of TIL therapy. In a recent randomized control study by Rohaan et al. it was demonstrated that in patients with advanced melanoma, PFS was significantly longer among those who received TIL therapy than among those who received ipilimumab although chemotherapy-related myelosuppression was noted among all TIL patients compared to 57% of patients on ipilimumab (79). A significant portion of the toxicity is due to high dose IL-2 infusio which can lead to hemodynamic instability and other high grade toxicities. An ongoing clinical trial to evaluate TILs modified with an inducible, membrane-bound IL-15 gene will explore whether IL-2 can be eliminated from the ACT protocol (NCT06060613 and NCT05470283), which, as stated above, could potentially significantly reduce the toxicity of the overall regimen. While TIL therapy is effective for treatment-refractory advanced melanoma, more work is required to improve efficacy, expand access to more patients, and apply TIL therapy to other solid tumors.

### Cytokine-induced killer cells

Cytokine-induced killer (CIK) cells are a unique polyclonal CD3 + CD56+ T-cell population with NK-like functional characteristics (80). Their MHC-unrestricted lytic ability allows them to target a broad range of tumors without prior antigen exposure, potentially making them more straightforward and more cost-effective than other therapies like CAR-T cells. CIK cells also have a reduced risk of GVHD. Clinical trials have shown the safety and efficacy of CIK cells in solid tumors, including lung cancer, hepatocellular carcinoma (HCC), renal cell carcinoma (RCC), and lymphoma (81). A phase III trial in HCC patients demonstrated a 14-month improvement in disease-free survival with mild to moderate adverse effects (82). Their low toxicity and feasibility have encouraged research into combination therapies, including anti-PD-1 immunotherapy, particularly for PD-1-resistant cancers. CIK cells can infiltrate tumors and secrete INF-*γ*, enhancing anti-PD-1 effectiveness, as seen in metastatic RCC (83) and non-small cell lung cancer (NSCLC) (84). Moreover, CIK cells have emerged as a platform for genetic modification with chimeric antigen receptors (CARs), enhancing their cytotoxicity in cancers like AML, ALL, and sarcomas. Clinical trials have shown that CAR-CIK cells maintain safety, with no GVHD or severe toxicities, even at higher doses, offering an advantage over CAR-T cells (85). The versatility of CIK cells makes them promising for combination therapies with chemotherapy, monoclonal antibodies, and bispecific antibodies, particularly in the treatment of solid tumors. While CIK cell immunotherapy has shown great potential, challenges remain in refining its precision and optimizing its clinical application.

### Macrophage targeting therapies

Tumor-associated macrophages (TAMs) are abundant in cancer lesions and contribute to the TME. They promote cancer cell growth by secreting chemokines and cytokines like IL-1α, IL-1β, and TNFα and inhibit CD8+ T cells by releasing immunosuppressive factors and triggering T cell apoptosis through FAS receptors, tumor necrosis factor-related apoptosis-inducing ligand (TRAIL). TAMs also contribute to cancer treatment resistance through mechanisms like epithelial-mesenchymal transition (EMT), tumor angiogenesis, and immune suppression. Nanomaterials combined with TAM-targeting therapies improve drug delivery and anti-cancer effects by polarizing macrophages into an anti-tumoral state (86). TAM-targeting therapies face challenges due to macrophage heterogeneity, systemic toxicity, and difficulties in the TME. Monotherapies often show limited success, but combining TAM-targeting with immune checkpoint inhibitors can enhance T cell function, block tumor growth, and reduce metastasis (87). CAR-M cells offer a novel therapeutic approach for overcoming solid tumor challenges. Studies have shown their ability to delay tumor growth and enhance anti-tumor immunity in mouse models of lung, ovarian, and pancreatic cancer (88). Ye et al. designed lipid nanoparticles (LNPs) that contain CAR mRNA and generate anti-CD19 CAR M by transferring LNPs to murine primary macrophages, demonstrating notable cytotoxic effects against human B-cell lymphoma *in-vitro* (89). Unlike CAR-T and CAR-NK cells, CAR-M cells resist exhaustion in the hostile TME and maintain anti-tumor function. However, barriers like “on target-off tumor” effects and tumor heterogeneity still limit the efficacy of CAR-M cell therapies (86). Clinical trials are ongoing (NCT04405778 and NCT05164666), which will likely provide more data to confirm their effectiveness.

### T-cell receptor therapies

TCR-based adaptive therapy involves genetically modified lymphocytes targeting specific tumor markers. Unlike CAR technology, which uses an artificial receptor to recognize tumor cell surface proteins, TCR-engineered cells employ a natural or slightly modified TCR to target tumor-specific epitopes presented by MHC molecules. This offers broader applicability, as more tumor-specific sequences exist within cells than on their surfaces (90). Afamitresgene autoleucel (afami-cel) recently received FDA approval for advanced synovial sarcoma (91). Several toxicities associated with TCR-T cells have been described in the clinic. On-target off-tumor toxicities are linked to target antigen expression in normal tissues and are primarily associated with TAAs. In clinical trials targeting MART-1 and gp100 with TCR-T cell therapy, ocular, cutaneous, and auditive toxicities were due to TAA expression in melanocytes. Primary resistance mechanisms may be mainly represented by a low or heterogeneous expression of the target antigen in tumor cells or by the tumor cells’ intrinsic resistance to T cell-mediated cytotoxicity (92). Key challenges include TCR product manufacturing, patient selection, and overcoming the immunosuppressive microenvironment to improve therapy efficacy and safety.

### Dendritic vaccines

Dendritic cells (DCs), the most potent antigen-presenting cells (APCs), play a key role in initiating and regulating both innate and adaptive immune responses (93). DC-based vaccines have emerged as a promising cancer immunotherapy, aiming to induce antigen-specific cellular immunity to eliminate cancer cells as they target a broader range of intracellular antigens compared to adoptive cell therapies (ACTs), which focus on tumor-specific surface antigens and face challenges like antigen escape and off-target toxicity (94, 95). Sipuleucel-T (Provenge) is the first FDA-approved therapeutic DC vaccine for metastatic castration-resistant prostate cancer, showing efficacy in phase III trials. Its combination with immune checkpoint blockers (ICBs) and IL-7 has shown encouraging clinical outcomes (Phase I, NCT01832870; Phase II, NCT01804465, NCT01881867) (96). Despite the success of sipulleucel-T, conventional DC vaccines have seen limited efficacy, benefiting only 5–15% of patients, likely due to immunosuppressive factors in the TME. To enhance antitumor responses, next-generation DC vaccines are being explored in combination therapies with ICBs to overcome TME-related challenges (97).

### Induced pluripotent stem cell derived immune cells

Induced pluripotent stem cells (iPSCs) are created by reprogramming adult cells to regain their pluripotent abilities. These cells can be modified to express CARs or TCRs for enhanced tumor specificity, offering the potential for more precise and effective cancer therapies. iPSCs can also be differentiated into NK cells, which are known for their ability to kill cancer cells (98). Genetically engineered iPSC-derived NK cells exhibit improved persistence and cytotoxicity, making them a promising cancer treatment (99). Additionally, iPSCs offer a renewable source for generating tumor-infiltrating lymphocytes (TILs) in large quantities, overcoming challenges related to the limited supply of TILs (100). iPSC-derived T cells, modified to express tumor-specific TCRs or CARs, can be expanded and infused into patients to mount an effective anti-tumor response (101). Despite their potential, challenges remain, including ensuring the safety of iPSC-derived immune cells, refining differentiation protocols, and overcoming cancer cells’ immune evasion. Overall, iPSC-based immunotherapies hold promise for personalized, targeted cancer treatments. Recent investigations have highlighted that CAR-NK cells generated from iPSCs not only significantly inhibited tumor growth but also extended survival in an ovarian cancer xenograft model (68, 102).

### Natural killer cells

In contrast to CAR-T cells, CAR-NK cells utilize another key component of the immune system that plays both an innate and adaptive role. NK cells function independently of the MHC and do not cause GVHD if an allogenic construct is used (47). In a clinical trial (NCT03056339) targeting CD19-positive lymphoid tumors, NK cells were transduced to express genes encoding anti-CD19 CAR, IL-15, and inducible caspase 9 as a safety switch. Out of the 11 treated patients, 8 (73%) responded, and 7 (64%) achieved complete remission. Notably, there were no cases of CRS or neurotoxicity, and no apparent increase in inflammatory cytokines or GVHD was observed with this HLA-mismatched CAR-NK product (67). This initial study underscores the safety advantages of CAR-NK cells in universal cell therapy.

## Regulatory pathways of immune effector cell therapies

The regulation of CAR T-cell therapies in the United States falls under the purview of the FDA, specifically overseen by the Office of Tissues and Advanced Therapies (OTAT) within the Center for Biologics Evaluations and Research (CBER) (103). It is important to acknowledge, that CAR-T cell therapy, primarily indicated for cancer treatment, undergoes Biologics License Application (BLA) review by the Oncologic Drugs Advisory Committee (ODAC). ODAC’s public meetings aim to enhance transparency, sharing safety data before approval, and it assesses safety and efficacy, voting on product approval based on the benefit–risk ratio (104).

The development of CAR T-cell therapy, originating in the 1990s, poses challenges in balancing efficacy and patient safety during first-in-human clinical trials. Recognizing the need for effective clinical trial protocols, the FDA introduced the (Initial Targeted Engagement for Regulatory Advice on CBER products) INTERACT program, offering informal advice on early investigational product development (105). A pre-IND (Investigational New Drug) meeting is crucial for CAR T-cell therapy development, providing an opportunity to refine strategies, identify necessary studies, and gain FDA insights within a rapid response timeframe. While CAR-T cells are categorized as regenerative medicine products in the United States, on a global scale, they are classified under the umbrella term of Advanced Therapy Medicinal Products (106, 107). This category encompasses gene therapies, as well as human cells, tissues, and products derived from them, all of which necessitate licensing. CBER released a series of regenerative medicine guidance documents in November 2017 to better define the regulatory environment for products related to regenerative medicine, including CAR-T cells.

The Regenerative Medicine Advanced Therapy (RMAT) designation programme which was established by the 21st Century Cures Act, was passed into law in December 2016, to expedite the development and approval of regenerative medicines, such as gene therapy. According to the Act if the preliminary clinical evidence suggests a potential to benefit unmet medical requirements for a significant or life-threatening disease or condition, regenerative medicine therapy may be eligible for RMAT certification (108). RMAT designation request, essential for expedited development, must meet specific criteria, and the IND submission involves comprehensive documentation, with a mandatory 30-day waiting period before clinical trials. A pre-BLA meeting, ideally 6 months before submission, addresses formatting, data adequacy, risk management, and safety studies. BLA submission undergoes a 10-month review, reduced to six for priority applications, and FDA’s response includes potential Advisory Committee meetings to address identified deficiencies and ensure thorough reviews CAR T-cell therapies, vital for treating life-threatening conditions with unmet medical needs, necessitate expedited regulatory approval, which often takes lengthy time periods for approval (109). The FDA has started several expedited programmes in recent years to shorten the processing time and facilitate the timely release of these rejuvenating therapies onto the market. “Fast Track Designation, Breakthrough therapy,” “Accelerated approval,” and “Priority review and Regenerative Medicine Advanced Therapy” are a few of these initiatives.

Accelerated approval expedites the review process based on surrogate endpoints, predictive markers for clinical benefit. Understanding the difference between surrogate and clinical endpoints is crucial for selecting the appropriate expedited pathway. Surrogate endpoints, like decreased tumor size, predict clinical benefit, while clinical endpoints, like longer survival, directly measure it (110, 111).

Fast Track expedites drug reviews for conditions currently lacking therapies or showing superiority over existing ones. It encourages frequent sponsor-FDA communication, fostering an efficient process for quicker patient access. Breakthrough Therapy is suitable for drugs demonstrating preliminary evidence on clinically significant endpoints. Submission deadlines for Fast Track and Breakthrough Therapy are pre-BLA, while Priority Review can be requested at BLA submission, with FDA responding within 60 days. This timeline does not apply to accelerated approval pathways (112).

Similar to CART, many other forms of IECs have been approved recently by FDA on accelerated means. In oncology, accelerated approvals have been extensively researched. Using ratings from worldwide health technology assessment reviews, one study specifically looked at the therapeutic value of cancer medications that were granted fast approval between 2007 and 2021. The study indicated that nearly 40% of U.S. fast approvals were classified as having high enhanced therapeutic value (113).

Since 2014, the FDA has approved more than 10 marketing applications for BiTEs to treat hematological and solid malignancies through the accelerated pathways (114). The FDA approves other immunotherapies like TILs, macrophage targeting therapies, dendritic vaccines, and iPSCs through a structured regulatory process through the same pathways discussed above for CAR-T therapy from submitting an IND application and being eligible for other accelerated programs such as Breakthrough Therapy, which is awarded to therapies with strong early clinical potential, and Fast Track, which encourages regular contact with the FDA.

### Regulatory pathways for immunotherapies outside the United States

Because distinct timetables and frameworks apply in the US, Europe, and China, there are differences in the regulatory procedures for immunotherapy approval. The FDA expedites market authorization for oncology therapies in the US by utilizing methods such as breakthrough therapy designation and accelerated approval, which result in considerably shorter review durations. Comparably, the EMA has its PRIME plan; yet, in both regions, the median review periods for new oncology medicines have increased, despite efforts to expedite processes (115). Additionally, the EMA runs a centralized system for Advanced Therapy Medicinal medicines (ATMPs), which include CAR-T cell therapies. These medicines are subject to hospital exemption restrictions for non-commercial, patient-specific goods and are examined by the Committee for Advanced Therapies (CAT). One permit is ultimately valid for all member states of the European Union (EU) as a result of this centralized review. The average review time has gone up, sometimes by 74 days, despite the EMA’s attempts to speed up procedures, such as conditional marketing authorizations (116, 117).

ATMPs are classified as “innovative biological products” by the National Medical Products Administration (NMPA), which is in charge of China’s immunological regulatory environment. By using tools like the Priority Review Pathway and Breakthrough Therapy designation, the NMPA has significantly accelerated the licensing process for immunotherapies, including CAR-T cell therapies. Consequently, China has decreased the time needed for new drug applications (NDAs), cutting the review timeframes to 200 days for regular applications and 130 days for those with priority review status (118). The NMPA also implemented parallel processing for inspections and reviews starting in 2020, which expedites the approval process even more. China has instituted an “implied license system,” cutting the review period for IND (Investigational New Drug approval) clearances from 90 days to 60 days, making it one of the quickest processes for advancing novel treatments into clinical trials (119).

## Economical aspects of immune effector cell therapies

### Cost factors associated with CAR-T cell therapy

Six CAR-T cell therapies have been approved by FDA since 2017, tisagenlecleucel for B-ALL under the age of 25 and LBCL, axicabtagene ciloleucel for LBCL, brexucabtagene autoleucel MCL, lisocabtagene maraleucel for LBCL, Idecabtagene vicleucel for MM, and ciltacabtagene autoleucel for MM.

On average, these therapies are priced at a one-time cost of $475,000 for B-ALL and $373,000 for B-NHL (120). The actual price can vary based on patient condition, healthcare system, and negotiated rates with the insurance companies. These high prices are secondary to the complex manufacturing process and administration of infusion therapy, but the actual price of the treatment can further increase, given the requirement of hospitalization, monitoring, diagnostic tests, and follow-up post CAR T-cell therapy. Given these large cumulative costs of treatment, nations, especially developing and underdeveloped nations, are facing a significant challenge to offer this as a treatment option to their patients. Even hospitals within the US healthcare system are reluctant to offer this treatment, at least in part due to complicated reimbursement policies. Previously, treatments like but not limited to gene therapy (e.g., voretigene neparvovec, onasemnogene abeparvovec-xioi, biologics/biopharmaceuticals, immunotherapies) (e.g., pembrolizumab, ipilimumab, nivolumab), and proton beam therapy have complicated the reimbursement process given their high costs. CAR T-cell therapy significantly compounds these challenges and adds to the difficulty for healthcare payers to get access to required treatments. Given the next generation CAR-T cell therapies are already in development, it may become exceedingly difficult to get them reimbursed, with the potential to exceed both the public and private ability to pay for these treatments. With 18% of American GDP going into health care, CAR-T will only add to that economic burden with its growing indications (121).

Expanding further on the complete financial costs of the CART therapy, the average sales price (ASP) of one of the most common therapies like tisagenlecleucel is around $529,192 as of October, 2024 data (122). ASP considers discounts, rebates, and other cost saving mechanisms available to healthcare payers. Tisagenlecleucel has a retail price of $612,745.39, meaning the retail and the ASP prices are comparable (123). This further means Medicare is more likely to cover this cost due to the structured ASP system with comparable costs. But this does not account for other parameters like inpatient stay costs, outpatient costs, costs of salvage therapy (mostly chemotherapy), and management of complications like CRS or neurotoxicity secondary to CART therapy. Hospitals administering CAR-T cell therapy must also account for expenses of training staff, certification and monitoring. Given the mean duration of hospital admission post-CART therapy is around 15 days, and the incidence of CRS is shown to be around 57 to 93%, the healthcare costs are quite high (124, 125). These prices can further increase if the patients require ICU stay post-CART therapy (125). A study showed that the total all-cause health care costs per patient, from 30 days prior to 90 days after infusion can be as high as $511,139 (125).

### Other therapies

Similarly, BiTE Cell therapies are also known to be expensive. The most common BiTE therapy, blinatumomab, given for relapsed or refractory B-ALL, has a point estimate ASP of $152.178 (122). The retail price of the medication is $5,427 per 35 mcg, and the overall cost can be as high as $178,000 to $250,000, depending on the number of cycles and dosage (126). As apparent, the current market for blinatumomab does not have an adequate structured ASP coverage system as per 2024, which can contribute to significant financial burden to healthpayers. Secondly, this does not account for the cost of inpatient stay/outpatient care and further complications associated with blinatumomab therapy. Further, studies have shown that patients who received blinatumomab have a probability of 40–83% to undergo hematopoietic stem cell transplantation in the future (127–129). This can have variable costs, depending on the study population, diagnosis, perspectives of the analyses, time horizons, and study methods of research (130). One study showed all-cause and HSCT-related costs up to $394,069 for patients with ALL in the US (131, 132). Adding the costs of subsequent salvage chemotherapy and cost of terminal care, the treatment prices can reach up to $700,000 to $1,000,000.

### Increasing markup pricing of CAR-T cell therapy

Medicare is the largest payer for cancer healthcare in the US and has chosen to cover CAR T-cell therapies. Medicare Part A is usually used with a payment system known as a Diagnosis Related Group (DRG). Given the cost of CAR-T cell therapy exceeds far more than what Medicare can pay (~$40,000), hospitals apply for outlier payment options through which, hospitals usually get ~$26,000 extra for the treatment. Additionally, hospitals apply for new technology add-on payments, especially available for new and expensive treatments like CAR T-cell therapies but this option only covers up to 50% of the market price of the drug (i.e., $186,500 out of $373,000). Hence, to get complete reimbursement and full extra payment, hospitals mark up the price of the therapy, thus increasing the price they charge for the drug. Even though these hospitals work within the framework of the Centers for Medicare and Medicaid Services (CMS) that establish guidelines and prevent fraud and abuse related to billing practices, the real price of providing the treatment is often unclear as it depends on many things discussed above. This creates ambiguity in the process, and hospitals can pay between less than $150,000 and sometimes more than $400,000 based on the marked-up price. The legality of marking up the cost of treatment is a complex issue and depends on various factors, including healthcare regulations, billing practices, and compliance with healthcare reimbursement laws. In the end, hospitals have the flexibility to not utilize marked up prices and provide the therapy at or near cost. That would increase access to this treatment while promoting transparency and cost contamination. Another option is opting for a site-neutral reimbursement, which gives clinicians more flexibility in choosing the setting in which they want to administer treatment. Patients and physicians would have an option to choose for the most cost-effective place, while ensuring that they get reimbursed equitably for their services. Considerations include hospitals that might face financial challenges, especially the ones that heavily rely on reimbursement rates for inpatient care, quality and continuity of care, and access to specialized care given complications post-treatment. Ultimately, this is a form of price discrimination which exists throughout the healthcare system and further transparency of cost is required to better control costs.

## Logistics of immune effector cell therapies

Besides economics, the major challenge with access to CAR-T cell therapy is the logistics of the cryogenic supply chain. CAR-T cell therapy access challenges can be divided by creating sub-sections within the vein-to-vein window. The vein-to-vein window is defined as the timeline between the T-cell collection from the donor and the transfusion of the manufactured product to the recipient. In the case of FDA approved CAR-T cell therapy at this time, the donor and recipient are the same, which complicates the logistics, unlike allogenic transplantations used in stem cells, which gives us the flexibility to transfuse stem cell products from one donor to multiple recipients. Allogenic CAR-T cell therapy products are currently being studied and even though they may increase the incidence of adverse events associated with this therapy, namely by the potential for GVHD, it would make CAR-T cell therapy more readily available to recipients with “off the shelf” availability. While the current infrastructure may be sufficient for the existing demand for CAR-T cell therapy, due to public expectations and the expansion of CAR-T cell therapy beyond R/R hematologic malignancies, and the recent approval of CAR-T cell therapy for highly prevalent neoplasms like multiple myeloma, which accounts for 1% of total cancers and is the second most common hematologic malignancy after lymphoma, the challenge has become to scale up manufacturing while expanding on cryogenic logistic networks (133).

### Understanding supply chain logistics of CAR-T cell therapy

The major challenge with the supply chain of CAR-T cell therapy is maintaining the-120 degrees Celsius (C) temperature of the biological sample during transportation. This temperature is required to safeguard the mobility of the sample, preventing its degradation and allowing its preservation for longer durations (134). The cryogenic transport can be divided into two parts, from patient to manufacturing team (collection sample) and from manufacturing team to patient treatment (manufactured sample), requiring adequate cryogenic preservation. This cryogenic cold packaging, also called dry shippers, requires several layers of packaging as a contingency plan to prevent rapid thawing and, thus, degradation of the biological sample. Beyond that, each package has a data logger and a smart monitor to measure the real-time temperatures of the package. Given that these dry shippers are one time manufactured to hold the sample and cannot be refrozen in case the temperature starts going above −120°C, these shippers undergo rigorous testing to fulfill the GMP standards and GDP requirements (135). These standards ensure that the samples are authorized for distribution while being transported under the correct conditions and prevent contamination of the samples. Here, the concept of hold-time comes in, measured by 24-h evaluation validation of the substance used in the dry shippers. As most of these dry shippers use liquid nitrogen (LN2) as a substance media for transport, a suitable volume of LN2 is used to maintain temperatures of −150°C to have a sufficient window for error below the critical threshold of −120°C while increasing the hold in time. Even with these measures, the variability of evaporation rate between dry shippers, difficulty in determining the amount of LN2 stored, inability to maintain vacuum seal, zeolite sponge degradation, and even tilting of the sample can change the hold time of the dry shippers, potentially creating a break in the supply chain logistics (136). There has been the recent development of LN2-free systems that use electrically powered cryocoolers, bypassing many of these possible errors while maintaining temperatures below −190°C (137).

These logistic problems coupled with physical, data integrity, and regulatory risks, can be addressed and improved upon in a number of ways. Rather than having a physical document trace of the manufacturing shipment at every transit location, cloud-connected monitoring and real-time alert systems should be in place for easy GPS tracking of the system and continuous temperature monitoring (138). This would help with the digitalization of the data, maintaining the data integrity across transport sites. Every sample should be GDP-certified with personnel trained in handling cryogenic logistics. Tilting of the samples can be minimized by bolting the samples to a large shipping pallet. Global standard operating procedures regarding sample handling requirements should be in place. At the clinical site on delivery, appropriate infrastructure for temporary cryogenic storage should be in place, and backup dry shippers should be put in place as a contingency plan. In the end, working with partners experienced in cryogenic transportation is necessary to scale up this technology to reach the masses.

Furthermore, a major challenge that promptly prevents access to CAR-T therapy are the limited number and location of manufacturing facilities and the distribution in inpatient tertiary and quaternary care centers (139). Expanding CAR-T cell therapy to community-based hospitals and outpatient clinics can increase availability and provide easy patient access. When required, the insurance providers’ slow prior authorization process for CAR-T cell therapy can be a further barrier to care (139). As the patients eligible for CAR-T cell therapy are the ones who have failed multiple previous treatments, these patients tend to be more ill and deconditioned than the general population having cancer. For these patients, prior authorization can take weeks to months for completion, which can either make these patients ineligible for CAR-T cell therapy due to worsening organ function or even death. Streamlining the prior authorization process while involving third-party companies to expedite the prior authorization may expedite this process, as saving every day for these patients is crucial to ensure they receive therapy in a timely manner (140). Moreover, prior authorization often requires a patient to undergo a series of diagnostic tests to determine the eligibility of the patient thereby often further restricting the access to CAR-T cell therapies. These requirements are expensive and require careful management of each step of prior authorization by the payer. In addition, the CMS recently established that insurers providing plans under the Medicare Advantage program can establish a “fail first’ therapy that requires Medicare patients with cancer to try less expensive therapies before qualifying for CAR-T cell therapy (141).

Other IEC therapies also require colder temperatures for storage and transport to prevent the degradation of these therapeutics. BiTEs are off-the-shelf biologics manufactured using recombinant DNA technology (130). They require refrigeration for storage at temperatures of 2–8°C (132). Similarly, antibodies from Macrophage-Targeting Therapies are usually stored at similar temperatures of 2–8°C and require a cold chain transport (131). This is because temperatures of 2–8°C are enough to maintain structural integrity and biological activity of antibodies and recombinant DNA’s. Storing them at temperatures of less than −150°C can cause cold denaturation and aggregation, compromising their efficacy (142). Whereas other immune cell effector therapies like TILs, Natural NK Cells, CIK Cells, engineered macrophages, dendritic vaccines, iPSC-derived IECs and TCR therapies are all living cells, and thus require storage temperatures of-150C and cryogenic rather than cold chain transport (143). They require the use of cryoprotective agents like dimethyl sulfoxide (DMSO) that prevents ice crystal formation and thus protects cellular structures. All of these therapies have similar key logistic challenges, namely complex manufacturing processes, regulatory and quality control issues, cost and scalability, supply chain infrastructure, especially the ones requiring cryogenic storage and transport, need for trained personnel and coordination between the centers so that the therapies get delivered on time.

### Ethical considerations

Given the economic and logistical constraints associated with the CAR T-cell therapy, key ethical challengers arise. These ethical challenges can be divided into pre-market and post-market, based on the patient population recruited in clinical trials, the development of medication, and the provision of equitable care.

### Patient recruitment

Patient recruitment includes following ethical principles and guidelines to recruit patients in clinical trials, developing appropriate informed consent strategies and managing adverse events. The key reason why an ethical dilemma arises in the pre-market phase is due to limited availability of slots in clinical trials as compared to the patients enrolled/waitlisted. A study done in Canada emphasized that in 2018, only one out of the three hospitals in Canada provided CAR-T services in the county, which has a population of 37 million (144). Additionally, only one manufacturing slot was allotted per month due to manufacturing limitations and there was no commercial manufacturing site available in Canada. Even with improved manufacturing capacity, there is a wait time of 4–6 weeks for apheresis and manufacturing, which is quite long given the patient population with R/R hematologic malignancies with an aggressive disease biology. Even through conditions have improved in recent years, ongoing expanding indications of CAR-T cell therapy with the recent approval of constructs in earlier lines of therapy and for more prevalent malignancies, including multiple myeloma, there has been a surge in the patients waiting for treatment. There is also a substantial expectation associated with CAR-T cell therapy through patients and their families, that the stakeholders, clinicians and manufacturers are trying to manage by accurately communicating the status of clinical research and potential risks associated with the therapy (Gene Cell Therapy Insights). Furthermore, there has been a surge in these medications for “compassionate use,” where manufacturers are requested to provide medications through the clinical trial process but not yet approved for general use as a standard of care therapy. There have also been many off-label requests submitted by patients and their advocates for the patients who do not fulfill the inclusion criteria for clinical trials. A recent multi-center study done in the US, which included 17 centers in the United States offering CAR-T cell therapy showed that since the FDA approval of idecabtagene for multiple myeloma, the median number of patients per center on the waitlist was 20 (range 5–100) and they remain on the waitlist for a median time of 6 months (range 2–8 months). Among these 17 centers, 25% patients died while waiting for their turn to receive treatment (80). Given the underlying demand not being able to meet the supply for the above-mentioned reasons, it is imperative to have ethical procedures in place to justify the equitable use of this novel therapy. The study done in Canada recruited a multidisciplinary cell therapy team including researchers, disease group leaders, and learners, patients, and other personnel with an operational or bioethical roles in the CAR-T processes (144). Accountability for Reasonableness (A4R) was used by this panel to identify the criteria of prioritization of patients to receive CAR-T therapy. After detailed discussion, four emergent themes were finalized, based on their decreasing level of weight. This included if the intent of therapy had curative potential, safety/risk of complications, psychosocial factors like non-compliance/lack of adherence, or medical urgency for treatment. Further, they shared the medical benefits assessment tool and safety/risk and non-weighted psychosocial assessment tool they used to classify patients (144). Similar results were seen in the multi-center study done in the US on patients with MM, where the most weight was given to the patients who would benefit the most from the CAR-T cell therapy (145). The goal is to ultimately make the recruitment process more standardized, as before the use of guidelines, the decisions were primarily based on perceived medical needs. This complicated the patient selection process as prioritization was at times largely influenced by the vigor of patient advocacy rather than guidelines. Currently, the ethical guidelines vary from institute to institute and the eventual goal would be to have a point-based system for CAR-T cell therapy, just like we use the United Network for Organ Sharing (UNOS) for organ transplantation. The UNOS is a point- based system for organ allocation in the USA which allows priority to the worst and maximizing total benefit (145). Unfortunately, reaching a universally agreed upon consensus across institutions nationwide will remain a challenge.

### Provision of equitable care

Given its high cost and geographical constraints and limited availability throughout the US, CAR-T cell therapy poses significant challenges in terms of healthcare disparities. Even though the average one-time cost of CAR-T cell therapy ($373,000–$475,000) has been considered reasonable by health economy assessments as per Incremental Cost-Effectiveness Ratio (ICER) and National Institute for Health and Care Excellence (NICE), this cost does not include handling toxicities post-treatment, inpatient stay, and regular follow-up investigation (146, 147). In the registrational trials for tisagenlecleucel, all patients experienced at least one adverse effect, with 84% (57/64) experiencing grade 3 (severe) or above adverse effects due to CAR-T therapy (7). The registrational trials for axicabtagene ciloleucel showed similar results with all patients having at least one adverse effect, and 94% (102/108) patients having grade 3 or above adverse effects (185). The most common of these adverse effects was CRS, happening in 79 and 94% patients, respectively, for tisagenlecleucel and axicabtagene ciloleucel. Managing these complications often warrants further in-hospital admission for the administration of CAR-T cell therapy, further adding to the cost (148). As a result, the estimated total cost of CAR-T cell therapy is $1.2 million (149). The limited number of sites offering CAR-T cell therapy also significantly adds to the economic burden, with out-of-pocket expenditure on transportation and prolonged lodging (as patients usually need to stay close to the site for at least 2–4 weeks post-administration) (150). These high costs primarily ensure access to CAR-T cell therapy solely for the wealthy and well-insured. Inadequate insurance coverage limiting the ability to pursue CAR-T cell therapy is a pivotal example of therapy unfairly stratified on socioeconomic terms. Given the high resource requirements of this therapy, its access is even further limited in developing/under-developed nations. Manufacturers should ensure that need/potential benefit should come first rather than the ability to pay. While many private health insurance plans will cover part of the cost for CAR-T cell therapy, the plan may not include deductibles and other expenses to be paid out-of-pocket expenses. The situation for public funded insurers is similar. Medicare will cover the cost CAR T-cell therapy based on the standard of care indication, while in contrast Medicaid covers it in only few states (141).

## Disparities in patient’s access

The widespread availability of CAR T cell therapy, despite recent advancements, is hindered by various challenges, notably impacting minority groups and those from lower economic backgrounds.

Disparities in patient’s access are evident across different cancer types. Multiple studies and recent analysis of 126 clinical trials on DBCL with CAR-T and BiTE therapies revealed that only 8% of the participants were African American (AA) and 92% white predominant (151–153). Similar underrepresentation was shown among patients with MM. A study by Ahmed et al. evaluating disparities in CAR-T cell therapy recipients revealed significant underrepresentation of AA, Hispanic, and Asian patients in clinical trials. For instance, in trials for MM treated with CAR-T cell therapy, only 1% of participants were AA, despite representing 20% of myeloma cases. Hispanic representation was similarly low at 5.4%, while white individuals were overrepresented, comprising 65% of the MM patient cohort (153). Karmali et al. had similar findings on patients with aggressive B-cell lymphomas. They revealed great underrepresentation of AA and Hispanics in this patient cohort as well (152). Additionally, Hadidi et al.*’s* observations indicated stark disparities affecting Black patients, with AAs constituting only 4% of DLBCL patients and 6% of MM patients (154). This underrepresentation is particularly concerning given the higher prevalence and earlier onset of certain cancers like MM in AAs and Hispanic populations, coupled with delayed access to innovative treatments (155, 156).

Consequently, these patients, already disadvantaged in terms of cancer incidence and mortality, face additional challenges in accessing advanced treatments. One explanation for this is that geographic distribution of clinical trials for CAR T-cell therapy, which did not include 60% of the states with the highest proportion of AA residents (157). Recently approved therapy of the TIL, lifileucel, is only available in 30 centers in the US. Some of the therapies such as CIK or NK cell therapies are mostly limited to early-stage clinical trials or available outside of the US (158). Other barriers include research mistrust, elevated trial participation costs, and restrictive enrollment criteria (159, 160). These findings underscore the need for more efforts to understand and address the lower rates of CAR-T cell therapy among minority groups.

Moreover, socioeconomic factors play a pivotal role in determining access to CAR T-cell therapy. Key elements include insurance coverage and the associated out-of-pocket costs, which can be prohibitively high. The expense of CAR T-cell therapy, ranging between $373,000 and $475,000, can be unaffordable for many, particularly those lacking adequate financial support or comprehensive insurance coverage (161). The mentioned above, lifileucel, will cost $515,000 according to manufacturer. Other expenses related to effector cell therapies were ranging between $373,000 to $475,000 and $72,000 for CAR T-cell and BiTEs therapies, respectively. Even though, the latter may seem to be more affordable option, it can be as costly as CAR T-cell therapy over time, especially for patients in long-term remission. The need for transplant consolidation, often excluded from short-term cost analyses, further adds to the expense (162, 163). The extent of insurance coverage, especially the distinction between private and public coverage, remains in need of increased transparency. Furthermore, there is a notable lag in insurers and governments covering new, cost prohibitive therapies like CAR T-cell therapy. For instance, although tisagenlecleucel was approved in the United States in August 2017, Medicare did not issue a national coverage determination until August 2019 (161). A major burden arises from out-of-pocket expenses, with distance and time to the nearest administering facility being a significant cost for transportation (164). CAR T-cell and BiTEs therapies are available in less than 4% of U.S. healthcare facilities, predominantly large academic centers and with less than 11% in non-urban environment (165). It was also shown that only 10 out of 31 states in the US had open CAR-T or BiTE trials (151, 166). Snyder et al. discovered that out of 3922 patients qualified for CAR T-cell therapy, over 37% had to travel for more than an hour to reach the closest academic hospital. These centers must undergo a rigorous onboarding process, including staff and patient education about the therapy’s significant toxicities, as part of a risk evaluation and mitigation strategy enrollment (167). These safety monitoring processes, while essential, can lead to substantial out-of-pocket expenses. For example, one study showed an average cost of $5,368 for both caregiver and patient, with higher costs ($7,191) for those in rural areas (164). This financial burden significantly limits access to CAR T-cell therapy. Ahmed et al.’s study revealed that only 7.3% of CAR-T cell therapy-related admissions were from patients in neighborhoods with an average income below $40,000 (153). Nearly one-third of CAR-T cell recipients lived more than 2 h away from the treatment center, primarily from higher socioeconomic backgrounds. The study also found fewer Medicare and uninsured patients in the CAR-T therapy group, highlighting insurance coverage as a significant determinant of therapy access (153). To overcome these obstacles, multiple strategies, such as increasing the number of sites offering CAR-T cell therapy and expanding local lodging, food, and transportation provisions for minorities are necessary.

A 24-question survey done in 2023 (TACTUM-23) which involved referring-center oncologists and treating-center oncologists for CAR-T therapy and BiTEs for the treatment of multiple myeloma (168). This survey showed that the top barriers to CAR-T cell therapy were the number of production slots (69%), followed by insurance/financial toxicity (15%), hospitalization requirements (8%), and manufacture time (8%). Similar responses for the top barrier to BiTE’s was hospitalization requirements (63%), toxicity monitoring requirements (13%) and, weekly dosing at specialized centers (17%). Proposed solutions include allogenic products, decentralization and point-of-care production of these therapies, developing all outpatient regimens, and rapid manufacturing platforms. Regardless, the American Society for Transplantation and Cellular Therapy recommends a referral to these centers for patients with cancer recurrence (169).

Nonetheless, many therapies have shown economic promise compared to other standard therapies currently in the market. Cost-utility analysis in an open-label, randomized phase 3 clinical trial comparing TILs and ipilimumab for unresectable melanoma showed total lifetime undiscounted costs of €347,168 for TIL therapy (including the production costs) and 3.52 quality-adjusted life years as compared to €433,634 and 2.46 QALYs for ipilimumab (79). Another study showed a dominant incremental cost-effectiveness ratio favoring TILs as compared to ipilimumab, adding to the previous literature (79). Similarly, studies have shown that the median cost for patients receiving induction and consolidation therapy for AML was € 32,648 (range: € 4,759–€ 140,383). These costs go up to € 88,635 (range: € 23,392–€ 215,119) when standard therapy is followed by immunotherapy using dendritic cells engineered to express the Wilm’s tumor protein is added to the regimen (170). DC vaccines are more complicated to manufacture secondary to the requirement of antigen loading, which is unlike that of CIK cells, iPSC cells, and NK cells (171). Regardless, limitations like *ex vivo* generation, quality control, transportation, storage, culture systems, regulatory compliance, and scalability continue to be significant logistical limitations to all immune cell effector therapies (Table 1).

**Table 1:** Summary of FDA approved immune effector cell therapies.

ICE	Product	Year approved	Target	Clinical trial	Efficacy	Safety profile		
					Overall survival	Progression free survival	Grade 3 or higher CRS	Grade 3 or higher neurologic events
CAR-T Cell	Axicabtagene ciloleucel (axi-cel)	LBCL-2022 Relapsed LBCL- 2017 Relapsed FL- 2021	CD19	ZUMA-1 (185)	52%	5.8 months	13%	28%
				ZUMA-5 (186)	NR	40.2 months	7%	19%
				ZUMA-7 (187)	61%	14.7 months	6%	21%
	Tisagenlecleucel (tisa-cel)	LBCL- 2018 FL- 2022 R/R B-ALL- 2021	CD19	BELINDA (188)	NR	3 months	5.2%	1.9%
				ELARA (13)	NR	NR	48.5%	37.1%
				JULIET (17)	49%	65%	22%	12%
				ELIANA (15)	76%	50%	77%	40%
	Lisocabtagene maraleucel (lisa-cel)	Relapsed LBCL- 2021 Refractory LBCL-2022	CD19	TRANSCEND (19)	18.8 months	NR	2%	10%
				TRANSFORM (189)	NR	10.1 months	1%	4%
				PILOT (190)	NR	NR	1.65%	4.89%
	Brexucabtagene autoleucal (brex-cel)	R/R Mantle Cell Lymphoma- 2020 R/R B-ALL- 2021	CD19	ZUMA-2 (191)	46.6 Months	25.8 Months	12%	20%
				ZUMA-3 (192)	12.1 months	NR	33%	11%
	Idecabtagene vicleucel (ide-cel)	RRMM-2021	BCMA	KarMMa (16)	19.4 Months	8.8 months	5%	3%
				KarMMA-3 (12)	12.4 Months	13.3 Months	5%	3%
	Cilatacabtagene autoleucel (cilta-cel)	RRMM-2022	BCMA	CARTITUDE-4 (193)		75.9%	1.1%	2.8%
				CARTITUDE-1 (194)	89%	77%	4%	9%
BiTEs	Blinatumomab	Refractory B ALL- 2014	CD3 and CD19	TOWER (195)	7.7 months	31%	4.9%	9.4%

Emicizumab-kxwh	Hemophilia A with factor VIII inhibitors- 2017	Factor IX and X	HAVEN 1, 2, 3	87% difference in bleeding events	NR	NR	NR

Amivantamab-vmjw	Metastatic small cell lung cancer- 2021		MARIPOSA (196)	NR	23.7 months	NR	NR

Tebentafusp-tebn	Metastatic uveal melanoma- 2022	anti-CD3 effector, glycoprotein 100–positive cells	NCT03070392 (73)	73%	31%	1%	NR

Faricimab-svoa	Age related macular degeneration- 2022	VEGF-A and angiopoetin-2	TRUCKEE (197)	BCVA +1.1 Mean CST reduction −31.3uM	NR	NR	NR

Teclistamab-cqyv	RRMM- 2022	CD3	MajesTEC-1 (198)	18.3 months	11.3 Months	0.6%	0.6%

Mosunetuzumab-axgb	RRMM- 2022	CD3 and CD20	NCT02500407 (199)		11.8 months	1%	4.1%

Epcoritamab-bysp	RR DLBCL, 2023	CD3 and CD20	NCT03625037 (200)		4.4 months	2.5%	0.6%

Glofitamab-gxbm	RR DLBCL 2023		NCT03075696 (201)		4.9 months	4%	3%

Tarlatamab	Small cell lung cancer-	delta-like ligand 3 and CD3	DeLLphi-301 (114)	14.3 months	NR	97–100%	58–65%
	Elranatamab		B-cell maturation antigen (BCMA)-CD3 bispecific antibody	Magnetis MM-3 (202)	12.4 months	4.6 months	0	0
TIL	Lifileucel	Advanced melanoma- 2024		C-144-01study (203)	13.9 months	4.1 months	0.6%	0.6%

## Global disparities in access to immune effector therapies

CAR T cell therapy remains to be the most common IEC; however, its global availability remains significantly constrained, despite its initial FDA approval for specific malignancies in 2017. While the adoption of CAR T-cell therapies has risen in the US, Europe, and China, global access remains scarce. This limitation is likely influenced by various factors, some intrinsic to the therapy itself and others specific to individual regions or countries (166). Previous investigations have identified critical barriers to the global adoption of CAR T-cell therapies, including patient eligibility, the scarcity of treatment centers, economic considerations, logistical challenges, manufacturing constraints, regulatory hurdles, and a lack of expertise in managing therapy-related toxicities (166).

The current available CAR-T cell therapies have usually been limited to large academic centers, thus this creates a lack of access due to geographical barriers, that vary widely among countries, as patients need to reside close to the respective academic center to get an effective treatment (139). An example of the wide variation of geographical constraints among countries can be elucidated by contrasting, a prior study in Peru, that described higher mortality rates in provinces with prolonged travel time to a healthcare facility among pediatric patients with leukemia (172). Whereas, in the US, it is estimated that 46.7% of the population resides within 30 min of a transplant center and 93.9% of the population can reach a transplant center within 180 min, bearing in mind that most of the CAR-T cell therapies are available in those facilities (173). An interesting approach to improve the geographical barriers is the implementation of CAR-T-capable outpatient centers, given the growing evidence that supports its safety when administrated in the ambulatory setting (174).

Furthermore, clinicians’ perceptions regarding CAR-T cell therapy’s availability must be considered. For instance, a recent survey conducted by Atallah et al. revealed that, in the context of CAR T-cell therapy for refractory MM, physicians in France, Morocco, and Saudi Arabia reported easy access to novel CAR T-cell therapies (175). Additionally, The Asia-Pacific Blood and Marrow Transplantation Group reported that CAR-T cell therapy was accessible in only 9 out of 19 countries/regions represented by their participants, with nearly half of them available solely through clinical trials (161, 176).

An additional significant impediment to the widespread adoption of CAR-T cell therapy is the overall dearth of decentralized research. Most clinical trials are conducted between the US, Europe, and China, with minimal activity in India, Mexico, Brazil, and Argentina, and none in Africa (1, 8). Kumar Suvvari et al., reported that the CAR-T cell therapy trials are already limited due to factors, namely high cost, and technical requirements (177). Notably, it is not surprising that these limitations are more pronounced in other countries, especially middle and low-income ones, where the available research funds are reserved for other country-specific priorities, and CAR-T cell therapy is not classified as essential or cost-effective at this time (164, 166). Therefore, it is expected that initially the availability of CAR-T cell therapy will be restricted to the private sector in many countries, worsening the economic disparities (166, 178).

Remarkably, a recent study by Elsallab et al. demonstrated that the current demand for autologous CAR-T cell products has surpassed the production capacity of centralized manufacturing sites, further limiting global access (166, 179). Improvement in access is expected in the near future as India is currently undergoing a phase III clinical trial with a strategy to manufacture CAR-T cells and reduce the cost of the therapy while increasing the availability to those in need worldwide (180).

It is imperative to undertake worldwide efforts aimed at enhancing research, accessibility, and manufacturing of CAR T-cell therapy to address global disparities, enhance patient outcomes, and advance the development of superior immune effector therapies. A good example of this is the creation of interest groups that advocate for access to CAR-T cell therapies, like the Global Gene Therapy Initiative (GGTI) or the Latin American Consortium collaboration with Caring Cross (181).

Regarding, other IEC therapies, like CAR-NK, TCR or TIL therapy. It has been proposed that the CAR-NK therapy could be more adaptable to different contexts, given its off-the-shelf availability, safety and manufacturing cost (182). However, more research is needed to evaluate its adaptability, and cost-effectiveness outside the US. Specially as most of the current global clinical trials, and governmental efforts are focused only on CAR-T cell therapy, and/or exclude other IEC therapies (183, 184).

## Conclusion

We conclude with summary of the complex social, economic, logistical, and regulatory challenges restricting access to IEC therapies. Despite expedited FDA programs, regulatory processes often cause harmful delays for urgent treatments. Economically, the high costs of manufacturing and infrastructure create significant barriers, with treatment prices posing severe financial challenges. Inconsistent insurance coverage, a lack of pricing transparency, and high out-of-pocket expenses further limit access.

Logistically, maintaining ultra-low temperatures during transportation is crucial but challenging. Limited manufacturing facilities and treatment centers, primarily in large academic institutions, restrict access for rural and underdeveloped regions. Expanding therapy to community hospitals and outpatient settings and streamlining prior authorization processes could improve accessibility.

Socially, minority groups and lower socioeconomic backgrounds face pronounced disparities. Studies highlight the underrepresentation of AA, Hispanic, and Asian patients in clinical trials. Efforts to improve access must include increasing treatment centers, enhancing patient and caregiver support, and ensuring transparent insurance policies.

The field is rapidly evolving, with ongoing research and expanding indications promising improved outcomes and broader access. Furthering global collaborative efforts between healthcare institutions and IEC manufacturers are crucial to addressing these barriers and ensuring cellular therapy reaches all eligible patients.
